# Reference Values of Thromboelastometry Parameters in Healthy Term Neonates Using NATEM in Cord Blood Samples

**DOI:** 10.3390/children9010047

**Published:** 2022-01-02

**Authors:** Alma Sulaj, Marina Tsaousi, Eleni Karapati, Abraham Pouliakis, Zoi Iliodromiti, Theodora Boutsikou, Serena Valsami, Nicoletta Iacovidou, Marianna Politou, Rozeta Sokou

**Affiliations:** 1 Neonatal Department, Aretaieio Hospital, School of Medicine, National and Kapodistrian University of Athens, 11528 Athens, Greece; alma_sulaj@hotmail.com (A.S.); marina_11@windowslive.com (M.T.); helenak5@hotmail.com (E.K.); ziliodromiti@yahoo.gr (Z.I.); theobtsk@gmail.com (T.B.); niciac58@gmail.com (N.I.); 2 2nd Department of Pathology, University General Hospital Attikon, School of Medicine, National and Kapodistrian University of Athens, 12462 Athens, Greece; apou1967@gmail.com; 3 Hematology Laboratory Blood Bank, Aretaieio Hospital, School of Medicine, National and Kapodistrian University of Athens, 11528 Athens, Greece; serenavalsami@yahoo.com (S.V.); mariannapolitou@gmail.com (M.P.); 4 Neonatal Intensive Care Unit, “Agios Panteleimon” General Hospital of Nikaia, 18454 Piraeus, Greece

**Keywords:** thromboelastometry, NATEM, neonates, coagulation, cord blood, reference ranges

## Abstract

*Background*: ROTEM assay has gained increasing acceptance as a method for rapid and specific coagulation pathway assessment. However, its use in the neonatal population remains limited since reference ranges have not yet been established. *Aims*: (1) to determine reference ranges for healthy term neonates of ROTEM parameters using non-activated assay (NATEM) in cord blood samples; (2) to assess whether delivery mode, gender, gestational age, birth weight and blood group (ABO and Rhesus) of the neonate, coagulation disorder and anticoagulant medication of the mother have an impact on NATEM parameters. *Methods*: NATEM assay was conducted in cord blood samples of 189 term neonates without any medical history. *Results*: Reference ranges (2.5th and 97.5th percentiles) are established for clotting time (CT), clot formation time (CFT), α-angle, clot amplitude at 5, 10 and 20 min (A5, A10, A20), maximum clot firmness (MCF), lysis index at 30 and 60 min (LI30, LI60, %) and maximum clot elasticity (MCE). Reference ranges for NATEM are CT 182–499 s, CFT 63–176 s, α-angle 58–78°, A5 28–52 mm, A10 37–61 mm, A20 42–66 mm, MCF 43–67 mm, LI30 97–100%, LI60 87–98% and MCE 75–203. Male neonates appear to be more hypocoagulable than females. *Conclusions*: We demonstrate reference ranges for healthy term neonates in NATEM assay that could be used as a reference group for future studies of neonates with an underlying pathology.

## 1. Introduction

Hemostasis in the neonatal period is an evolving process that starts in the intrauterine life and continues to undergo changes until adulthood. This age-related maturation is described by the term “developmental hemostasis”, first described by Maureen Andrews in the 1980’s and includes quantitative and qualitative differences in hemostatic factors [1,2,3,4,5]. In the neonate, the concentration and the activity of both pro-coagulant factors (II, VII, IX, X, XI, XII), and anti-coagulant proteins (protein-S and -C) are below adult values. As to the platelets, although their number is within normal adult values, they appear hyporeactive. The increased concentration and the large multimers of von Willebrand factor (VWF), the higher hematocrit and the large nucleated red cells in neonates counterbalanced this hyporeactivity. The fibrinolytic system is immature, with reduced plasmin activity and low levels of plasminogen inhibitor. Despite these differences, healthy neonates have a hemostatic balance, without bleeding or thrombotic tendency [6,7,8]. 

Conventional coagulation tests used in clinical practice, include prothrombin time (PT), activated partial thromboplastin time (aPTT), international normalized ratio (INR), PLT counts, and fibrinogen. These tests are performed in plasma and they do not reflect the clot dynamic, the platelet function and the fibrinolytic capacity. Therefore, they are not representative of the in vivo hemostatic procedure and this renders their prognostic value of detecting bleeding diathesis of limited value [9,10]. Viscoelastic whole-blood analyses include Rotational thromboelastometry (ROTEM^®^, Tem Innovations GmbH, München, Germany) and thromboelastography (TEG) system overcome these obstacles. They provide a graphical curve that represents the in-vivo interaction between platelets, coagulation factors, fibrinogen/fibrin, endothelium and overall the formation, stabilization and lysis of the clot. Hence, they do not only detect coagulopathies but they also identify the type of coagulopathy and could thus constitute a useful tool for diagnosis and treatment [11,12]. The first results are readily available within 30 min, offering a prompt bedside evaluation tool for the patient. Thromboelastometry has been studied in adults and children, especially in cardiac surgery, transplantation, trauma, extracorporeal membrane oxygenation patients and obstetrics [13,14,15,16]. As far as the neonates are concerned, available data are scarce. Their clinical use is not widespread, due to the lack of reference ranges for this age group. The majority of the existing studies focuses on specific medical conditions such as sepsis, intraventricular hemorrhage (IVH), and hypoxic ischemic encephalopathy treated with therapeutic hypothermia [17,18,19,20,21,22,23,24].

The primary aim of our study was to determine reference ranges for term neonates without any medical history of ROTEM parameters using non-activated assay (NATEM) in cord blood samples, which can be used in future studies. Secondarily, to assess whether delivery mode, gestational age (GA), birth weight (BW), sex and blood group (ABO and Rhesus) of the neonate, coagulation disorder and anticoagulant medication of the mother have an impact on NATEM variables.

## 2. Materials and Methods

**Figure 1:**
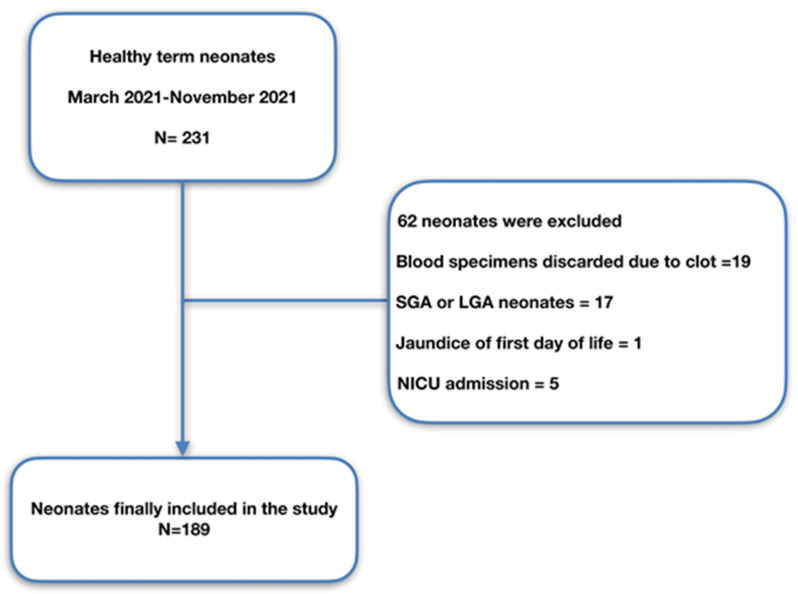
Study population flowchart. SGA, small for gestational age; LGA, large for gestation age; NICU, neonatal intensive care unit.

This is a longitudinal cohort study. Term neonates without any medical history with gestational age (GA) ≥37 weeks and appropriate birth weight (BW) for GA born at Magginio Maternity Clinic, Aretaieio Hospital, National and Kapodistrian University of Athens, from March 2021 up to November 2021 comprised the study population. The study was in accordance with all the relevant national regulations, and institutional policies and was authorised by the Institutional Review Board of Aretaieio Hospital, National and Kapodistrian University of Athens (Project identification code: 310/26-03-2021). Parental consent after a short briefing was acquired for all neonates included in the study. Data regarding GA, BW, gender, mode of delivery, Apgar score, blood group (ABO and Rhesus) of the neonates and pH of the umbilical cord were recorded. Data about maternal coagulopathy (such as MTHFR homozygosity, factor V Leiden heterozygosity, homocysteine homozygosity etc.) and use of anticoagulant medication were also documented. Neonates born by emergency cesarean section (CS), with a personal or family history of bleeding disorders, with a chromosomal abnormality or many dysmorphic features, sepsis, perinatal asphyxia, perinatal stress (defined criteria have been previously described [21]), gestational diabetes, with jaundice of the first day of life; small for gestational age (SGA), intrauterine growth restriction (IUGR), large for gestation age (LGA) and finally neonates admitted in neonatal intensive care unit (NICU) were excluded from the study. All neonates were followed up until their discharge. Data regarding recruitment of our study are presented in the flowchart (Figure 1). 

Cord blood samples were collected from the umbilical cord immediately after clamping using 21G needle and were then transferred into 3.5 mL 9NC Coagulation sodium citrate 3.2% containing VACUETTE^®^ TUBE. Blood samples with fibrin clots were discarded. Whole blood (300 μL) was analyzed on the ROTEM^®^ delta analyzer (Tem Innovations GmbH, Munich, Germany) using the NATEM assay. Immediately after collection, the sample was gently inverted five times to resuspend any sediment. It was then incubated for 2–5 min at 37 °C and was tested within 30–60 min. The ROTEM test was performed using the respective automated pipette programs according to the manufacturer’s guidelines. Clot formation was induced by adding 20 μL of 0.2 M calcium chloride solution (star-TEM ^®^ 20 reagent, Tem Innovations GmbH, Munich, Germany). The reagent was added to the cup and then was adequately mixed with 300 μL of whole blood anticoagulated with 0.109 mol/L trisodium citrate (9:1, v/v blood anticoagulant, Greiner Bio-One GmbH, Kremsmünster, Austria). The assay ran for at least 60 min after clot lysis at 30 min. The following NATEM variables were recorded: clotting time (CT, seconds), the time passed from the start of measurement until the formation of a clot 2 mm in amplitude; clot formation time (CFT, seconds), the time between 2 mm and 20 mm of clot amplitude clot amplitude at 5, 10 and 20 min after CT (A5, A10, A20); α-angle (a°), the angle between the central line (*x*-axis) and the tangent of the clotting curve at the amplitude point of 2 mm, reflecting the clot kinetics and measured in degrees; maximum clot firmness (MCF, mm), the widest amplitude of main body of trace; lysis index at 30 and 60 min (LI30, LI60, %), the percentage of remaining clot stability proportionate to the MCF following the 60-min observation period after CT indicating the speed of fibrinolysis and maximum clot elasticity (MCE = 100 × MCF / (100 − MCF)).

### Statistical Analysis

Statistical analysis was performed within the environment of SAS for Windows version 9.4 platform (SAS Institute Inc., Cary, NC, USA). Descriptive values were expressed using the median value and the relevant quartiles, i.e., Quartile 1 (Q1) to Quartile 3 (Q3) range. Comparisons among the groups for qualitative parameters were performed by the chi-square test (if the number of observations were less than 5 in more than 25% of the contingency table then the Fisher exact test was used). For continuous variables normality was not always possible to be ensured (as tested via the Kolmogorov-Smirnov test), therefore, non-parametric tests were applied; specifically we applied the Mann Whitney U test when comparing two groups and the Kruskal-Wallis test when comparing more than two groups. The significance level (i.e., threshold for the *p*-value) was set <0.05; moreover all statistical tests were two sided.

Calculation of reference values was performed using the value ranges and using non-parametric methods, as these: (i) are simpler to perform and (ii) do not require any assumption on data distributions. For the reference ranges, the 2.5 and 97.5 percentiles are reported, after controlling for outliers for each NATEM 

## 3. Results

One hundred and eighty-nine (189) term neonates without any medical history with median gestational age 39 weeks (Q1–Q3 = 39–40 weeks) were the study subjects. Forty-five (45) neonates out of them (23.8%) had GA between 37 and 38 weeks, 134 (70.9%) had GA between 39 and 40 weeks, and 10 had GA 41 weeks (5.3%). The median birth weight was 3330 g (Q1–Q3: 3140–3530) and 45.5% were females. Baseline characteristics of the study population are demonstrated in Table 1. 

All parameters of NATEM assay are depicted as median values and reference ranges (2.5th and 97.5th percentiles) in Table 2. 

Statistical analyses showed that GA had a positive, yet weak correlation with LI30 and LI60 (r = +0.193, *p* = 0.008, r = +0.191, *p* = 0.009 respectively), while no correlation was found between BW and NATEM parameters (*p* > 0.05). Male neonates appear to have a more hypocoagulable profile compared to females, expressed by the prolonged CT (*p* = 0.01) and lower A20 (*p* = 0.03), MCF (*p* = 0.03), MCE (*p* = 0.03) (Table 3).

Delivery mode had no impact on the NATEM parameters for all comparisons (CS, VD, FD, *p* > 0.05) (Table 4).

Blood group (ABO) had no influence on the NATEM parameters except for a minor impact on the LI60 for all comparisons (*p* = 0.046) (Table 5). Rhesus blood group had no impact on any NATEM parameter (*p* > 0.05). 

Regarding maternal coagulation disorders, neonates of mothers with thrombophilia appeared to have a more stable clot in 30 min, LI 30, (*p* < 0.05), although the difference seems to compensate in 60 min. Maternal anticoagulation treatment had no impact on NATEM parameters of neonates. 

## 4. Discussion

In this study we establish reference ranges for ROTEM variables of NATEM assay in cord blood samples of healthy term neonates. To our knowledge, we are the first to determine reference ranges for NATEM assay in cord blood.

ROTEM has recently gained acceptance as a point of care coagulation testing especially in perioperative assessment and blood transfusion guidance, as they provide a more thorough estimation of hemostatic cascade than standard coagulation tests do [4]. One of the most appealing characteristics of the method is real-time, rapid evaluation of the clot formation and degradation that requires a small quantity of blood [11,12]. Consequently, it constitutes a useful tool for diagnosis and treatment of coagulation derangement for all age groups, including the neonatal population [13,14,15,16,17,18,19,20,21,22,23,24]. However, the usefulness of ROTEM in neonates is not widespread, due to lack of reference ranges and standardization of the values, making the results difficult to interpret as they are usually compared with adults’. This comparison is though misleading owing to age-related differences in coagulation cascade i.e., developmental hemostasis [2,3]. Besides, the neonates cannot be considered “small adults”, taking in consideration the differentiation in the hemostatic system. 

The most commonly used ROTEM assays in patients with coagulation disorders are EXTEM/INTEM. They require the use of a reagent (recombinant tissue factor and polybrene/ellagic acid respectively) in addition to calcium; as such the results could be influenced and not precisely reflect the hemostatic status of the patient. There are studies, including patients, where EXTEM/INTEM parameters were within normal range although patients were bleeding actively [25]. On the other hand the NATEM assay is only activated by calcium without a supplemental reagent (star-tem ^®^ reagent). In this way, it could reveal hemorrhagic disorders more efficiently. It seems to be sensitive at detecting alteration in hemostatic profile of patients with sepsis, infection, trauma-induced coagulopathy (TIC), disseminated intravascular coagulopathy (DIC) and cirrhosis as it reflects the alteration in coagulation only affected by endogenous activator secreted by monocytes [26]. 

As far as we know the majority of the studies examining ROTEM in neonates have focused on other assays such as EXTEM, INTEM, FIBTEM, etc, except Sidlik et al. [27] who performed modified NATEM analysis with “an increasing concentration of tissue plasminogen activator (tPA)”, using though a smaller sample of 101 neonates. Comparing them to adult samples he reported an accelerated clot formation and fibrinolysis. No correlation was noted between modified NATEM parameters and gender, BW, Apgar score, delivery mode, maternal health conditions or medication. 

Oswald et al. [13] published age-related reference ranges for EXTEM, FIBTEM and INTEM. This study included distinct pediatric groups, showing an accelerated clot initiation and firmness in the neonatal subgroup. Theodaraki et al. [28] used the same assays including a bigger sample of 215 neonates, establishing reference ranges for that population. Sokou et al. [24] established reference ranges for term and preterm neonates with the use of EXTEM assay. Regarding reference ranges for TEG method, Liu et al. [23] had recruited the largest sample so far of 371 healthy full-term neonates.

In our study, NATEM parameters were influenced by gender. Males presented with a hypocoagulable profile in comparison to females, and had prolonged CT and lower A20, MCF and MCE, with all these values being statistically significant. Theodoraki et al. [28] found lower INTEM LI45 and LI60 values in males, showing an accelerated fibrinolysis, while those values were not gender-related in our findings. Data about sex-related differences regarding coagulation in neonates are insufficient. In accordance to our findings, Scarpelini et al. [29] and Ahammad et al. [30] noticed a hypercoagulable profile in female adults. The same correlation is also noticed in conventional coagulation tests, described by Sivrikaya et al. [31], with males having prolonged PT and INR. The aforementioned results could be explained by the known fact that females present with a higher level of coagulation factors. 

Although only full-term neonates were enrolled, a positive but weak correlation was established between GA and LI30, LI60. Theodoraki et al. [28] reported similar outcomes in INTEM and EXTEM assay. Regarding birthweight, no difference was noted in accordance to Sidlik et al. [27]. Mode of delivery did not influence our results in agreement with Schott et al. [32]. On the contrary, Liu et al. [23] observed a prolonged time of clot formation in neonates delivered by CS. In line with Theodoraki et al. [28], no correlation was found in ABO or Rhesus blood group and NATEM components, except a marginally statistically significant lower value of LI60 in AB blood group. Neonates born to mothers with thrombophilic disorders were noted to have a more stable clot at 30 min, although that was compensated in 60 min, with both groups having the same amount of clot lysis in percentage. Maternal anticoagulation treatment had no impact on our values according to the findings of Strauss et al. [17]. 

Nonetheless our study has limitations. First of all, since healthy term neonates without need of resuscitation consisted the subject of our study, delayed cord clamping or umbilical cord milking was a common practice. As a result, only small amounts of umbilical blood could be drawn and thus additional tests could not be performed, i.e., complete blood count, PT, aPTT that could be correlated with NATEM parameters. On the other hand, our large sample population (*n* = 189) allows confidence of the results at a level of 99%. In addition to this, it should be mentioned that the study was performed with blood samples from umbilical cord. According to current knowledge, cord blood seems equivalent to neonatal blood and could be used as an alternative, especially in preterm neonates in special clinical conditions such as sepsis evaluation [33,34,35]. However, especially when it comes to transfusion-guided therapy, data from umbilical cord samples should be interpreted with caution. Larger cohort studies are needed to verify our results and associate them with bleeding or thrombotic complications in ill or preterm neonates.

## 5. Conclusions

Our study has the largest sample size so far regarding cord blood NATEM values in healthy full-term neonates. We established reference ranges in NATEM assay that could be used as a reference group for future studies of neonates with an underlying pathology. Further studies could evaluate NATEM assay as a diagnostic and prognostic tool in neonatal coagulation disorders in order to provide optimal management. 

## Figures and Tables

**Table 1:** Baseline characteristics of the study population.

	Characteristic	*n*	Value *
Newborns’ data	GA (weeks) Median (Q1–Q3)	189	39 (38–40)
	BW (g) Median (Q1–Q3)	189	3330 (3140–3530)
	Gender (females)	189	86 (45.5%)
	Apgar score 1 min	189	9 (9–9)
	Apgar score 5 min	189	10 (10–10)
	pH (umbilical cord)	185	7.36 (7.33–7.38)
Newborns’ laboratory data	Blood group	183	A (62/33.88%) B (35/19.13%) AB (8/4.37%) O (78/42.62%)
	Rhesus	183	Positive (166/90.7%) Negative (17/9.3%)
Maternal data	Thrombophilic history	189	11 (5.82%)
	Anticoagulation treatment	189	36 (19.05%)
Pregnancy and delivery	Delivery mode	189	CS 116 (61.38%) VD 61 (32.28%) FD 12 (6.35%)

Abbreviations: CS, cesarean section; VD, vaginal delivery; FD, forceps delivery; * Measure is expressed as median and Q1–Q3 range, or as frequency and percentage.

**Table 2:** Reference ranges of NATEM parameters (*N* = 189).

Parameter	Median (Q1–Q3)	2.5 Pctl	97.5 Pctl
CT	322 (250–391)	182	499
A5	41 (36–45.5)	28	52
A10	51 (47–55)	37	61
A20	57 (53–61)	42	66
CFT	97 (80–127)	63	176
MCF	58 (54–61)	43	67
α-angle	71 (65–74)	58	78
LI30	100 (99–100)	97	100
LI60	93 (91–95)	87	98
MCE	136.5 (118–158.5)	75	203

Abbreviations: CT, clotting time (seconds); CFT, clot formation time (seconds); A5, A10, A20, clot amplitude at 5, 10 and 20 min, (mm); MCF, maximal clot firmness (mm); LI30, LI60, lysis index at 30 and 60 min (%); α-angle, alpha angle (°); MCE, maximum clot elasticity; NATEM, non-activated rotational thromboelastometry. N: Number of cases, Q1: first quartile, Q3: third quartile.

**Table 3:** NATEM parameters and gender of neonates.

Parameter	Gender		*p*-Value
	Males (*n* = 103) Median (Q1–Q3)	Females (*n* = 86) Median (Q1–Q3)	
CT	333 (280–413)	299.5 (245–370)	0.016
A5	40 (35–45)	42 (38–46)	0.105
A10	50 (46–55)	52 (49–55)	0.079
A20	56 (52–60)	58 (55–61)	0.035
CFT	103 (82–137)	92.5 (78–121)	0.071
MCF	57 (53–61)	59 (56–61)	0.032
α-angle	70 (64–74)	71 (67–74)	0.094
LI30	100 (99–100)	100 (99–100)	0.952
LI60	93 (90–95)	93 (91–95)	0.915
MCE	132.5 (114–157)	146 (127–159)	0.032

Abbreviations: CT, clotting time (seconds); CFT, clot formation time (seconds); A5, A10, A20, clot amplitude at 5, 10 and 20 min, (mm); MCF, maximal clot firmness (mm); LI30, LI60, lysis index at 30 and 60 min (%); α-angle, alpha angle (°); MCE, maximum clot elasticity; NATEM, non-activated rotational thromboelastometry; N: Number of cases, Q1: first quartile, Q3: third quartile.

**Table 4:** NATEM parameters and delivery mode.

Parameter	VD (*n* = 61) Median (Q1–Q3)	CS (*n* = 116) Median (Q1–Q3)	FD (*n* = 12) Median (Q1–Q3)	*p*-Value
CT	322 (232–377)	272.5 (321–401.5)	283.5 (236–378)	0.429
A5	42 (37.5–46.5)	36 (40.5–45)	42.5 (35.5–44)	0.389
A10	52 (47–56)	46.5 (50–54)	53 (47.5–54)	0.428
A20	57 (53–62)	53 (57–60)	59 (53–60.5)	0.413
CFT	88 (78–127)	81.5 (102.5–128)	113 (82–130.5)	0.232
MCF	59 (54–62)	54 (57.5–61)	59.5 (53.5–61.5)	0.408
α-angle	72 (65–74)	65 (70–74)	68.5 (66–73)	0.321
LI30	100 (99–100)	99 (100–100)	100 (99.5–100)	0.945
LI60	93 (91–95)	90 (93–94)	92 (91–94.5)	0.389
MCE	145 (120–162)	118 (135–154)	147 (115.5–161)	0.377

Abbreviations: CT, clotting time (seconds); CFT, clot formation time (seconds); A5, A10, A20, clot amplitude at 5, 10 and 20 min, (mm); MCF, maximal clot firmness (mm); LI30, LI60, lysis index at 30 and 60 min (%); α-angle, alpha angle (°); MCE, maximum clot elasticity; NATEM, non-activated rotational thromboelastometry; VD, vaginal delivery; CS, cesarean section; FD, forceps delivery; N: Number of cases, Q1: first quartile, Q3: third quartile.

**Table 5:** NATEM parameters and blood group of neonates.

Parameter	Blood Group ABO				
	A (*n* = 62) Median (Q1–Q3)	B (*n* = 35) Median (Q1–Q3)	AB (*n* = 8) Median (Q1–Q3)	O (*n* = 78) Median (Q1–Q3)	*p*-Value
CT	299.5 (246–377)	342 (255–415)	375 (303.5–420)	322.5 (250–385)	0.472
A5	41 (37–47)	39 (34–44)	43 (35–45.5)	42 (37–45)	0.306
A10	51 (48–57)	49 (45–53)	53.5 (46–54.5)	52 (47–54)	0.294
A20	58 (54–62)	55 (51–60)	59.5 (53–61)	57 (54–60)	0.169
CFT	92 (77–123)	109 (82–138)	102 (83–128.5)	97 (82–121)	0.284
MCF	59 (55–62)	56 (52–61)	59.5 (53.5–62.5)	58 (54–60)	0.194
α-angle	71.5 (66–74)	68 (63–74)	70 (65–73)	71 (67–74)	0.289
LI30	100 (100–100)	100 (99–100)	100 (99–100)	100 (99–100)	0.394
LI60	93 (91–95)	93 (90–94)	90 (90–92)	92 (90–94)	0.046
MCE	146 (121–166)	128 (109–157)	145 (113–162)	136.5 (120–153)	0.211

Abbreviations: CT, clotting time (seconds); CFT, clot formation time (seconds); A5, A10, A20, clot amplitude at 5, 10 and 20 min, (mm); MCF, maximal clot firmness (mm); LI30, LI60, lysis index at 30 and 60 min (%); α-angle, alpha angle (°); MCE, maximum clot elasticity; NATEM, non-activated rotational thromboelastometry; N: Number of cases, Q1: first quartile, Q3: third quartile.

## Data Availability

Date are available from the corresponding author upon a reasonable request.
